# Pre-Clinical Study of the [^18^F]AlF-Labeled HER2 Affibody for Non-Invasive HER2 Detection in Gastric Cancer

**DOI:** 10.3389/fmed.2022.803005

**Published:** 2022-02-16

**Authors:** Jingya Han, Yang Chen, Yan Zhao, Xinming Zhao, Jingmian Zhang, Jianfang Wang, Zhaoqi Zhang

**Affiliations:** ^1^Department of Nuclear Medicine, The Fourth Hospital of Hebei Medical University, Shijiazhuang, China; ^2^Department of Oncology, The Fourth Hospital of Hebei Medical University, Shijiazhuang, China; ^3^Hebei Provincial Key Laboratory of Tumor Microenvironment and Drug Resistance, Shijiazhuang, China

**Keywords:** human epidermal growth factor receptor (HER2), affibody, gastric cancer, molecular imaging, molecular probe

## Abstract

Human epidermal growth factor receptor 2 (HER2) is an important biomarker in gastric cancer (GC) and directly influences the therapeutic effect. Fluorine is firmly bound to Al^3+^ forming [^18^F]AlF-1,4,7-triazacyclononanetriacetic acid (NOTA)-HER2 affibody is a promising radiolabeled tracer that can monitor the changes of HER2 expression combining the advantages of simple preparation and the properties of ^18^F. The aim of this study was to develop a quick method for the synthesis of [^18^F]AlF-NOTA-HER2 affibody and evaluate its utility for HER2+ GC imaging in mouse models. Moreover, ^68^Ga-NOTA-HER2 affibody imaging was also performed to highlight the superiority of [^18^F]AlF-NOTA-HER2 affibody imaging in resolution. The HER2 affibody was conjugated with NOTA and labeled using ^18^F based on the complexation of [^18^F]AlF by NOTA. Its quality control and stability were performed by high-pressure liquid chromatography (HPLC). The molecular specificity and binding affinity of the novel radiotracer were evaluated in the GC cell line with HER2 overexpression (NCI-N87) and negative expression (MKN74). Distribution studies and PET/CT imaging were performed in mouse models. ^68^Ga-NOTA-HER2 affibody PET/CT imaging was also performed. [^18^F]AlF-NOTA-HER2 affibody was efficiently prepared within 30 min with a non-decay-corrected maximum yield of 32.69% and a radiochemical purity of more than 98%. [^18^F]AlF-NOTA-HER2 affibody was highly stable in incubation medium for 4 h *in vitro* and in the blood of nude mice at 30 min post-injection (p.i.). *In vitro* studies revealed specific binding and high binding affinity of the probe in NCI-N87 cells, while no binding was seen in MKN74 cells. PET imaging showed that NCI-N87 xenografts were differentiated from MKN74 xenografts with excellent contrast and low abdominal background, which was confirmed by the distribution results. High-level accumulation of the [^18^F]AlF-NOTA-HER2 affibody in HER2+ tumors was blocked by excess unlabeled NOTA-HER2 affibody. [^18^F]AlF-NOTA-HER2 affibody has a higher image resolution than that of ^68^Ga-NOTA-HER2 affibody. [^18^F]AlF-NOTA-HER2 affibody could be produced facilely with high radiochemical yield and may serve as a novel molecular probe with tremendous clinical potential for the non-invasive whole-body detection of the HER2 status in GC with good image contrast and resolution. This method could provide an *in vivo* understanding of GC biology that will ultimately guide the accurate diagnosis and treatment of GC.

## Introduction

Gastric cancer (GC) ranks fifth in terms of malignancy incidence and third in cancer-related deaths worldwide (1). In China, GC is one of the three most common cancers, and over 70% of patients with GC are diagnosed at an unresectable or metastatic stage (2). Moreover, primary or acquisition cytotoxic chemotherapy resistance renders GC with a very poor prognosis, and the median overall survival (OS) is 10–12 months (3). This finding emphasizes the urgency to identify new therapeutic targets to improve the prognosis.

Approximately 17–20% of patients with GC tested positive for the human epidermal growth factor receptor 2 (HER2) (4). The recommendation in the updated National Comprehensive Cancer Network (NCCN) and European Society for Medical Oncology (ESMO) guidelines suggested that all patients with unresectable, recurrent, or metastatic advanced gastric cancer (AGC) should be screened for HER2 positivity, and those with HER2+ tumors are eligible for first-line trastuzumab in combination with chemotherapy (3). Essentially, accurate monitoring of the HER2 status *in vivo* is critical for effective treatment. Gastroscopic biopsies are spatiotemporally limited due to the high heterogeneity of HER2 expression (5). HER2 heterogeneity may exist between primary tumors and recurrent focus, primary tumors and metastases, among different metastases, and even in the same lesion before and after treatment (6–11). Thus, accurate assessment of the HER2 status is critical in patients with metastatic or recurrent GC, even if the primary lesion is HER2– (12). Furthermore, a repeated biopsy is necessary to evaluate the efficacy of anti-HER2 agents during treatment because treatment resistance eventually develops in some patients with GC. The invasive biopsies are difficult to accurately reflect systemic lesions, monitor HER2 levels in real-time, and measure heterogeneity of tumors. Moreover, repeated invasive biopsies are not feasible in clinical practice considering the tolerance of patients. These limitations have contributed to the development of HER2-targeted molecular imaging and HER2 liquid biopsy technologies [ctDNA sequencing, ctRNA sequencing, circulating tumor cells (CTCs) detection, etc.] (13, 14). Thus, a whole-body, real-time, and non-invasive HER2-targeted molecular imaging method may be a potential alternative to biopsy-based methods for identifying patients suitable for HER2-targeted therapy and monitoring the therapeutic efficacy to improve the management of GC.

Many promising PET radionuclides with long half-lives and decay times, such as ^64^Cu, ^89^Zr, and ^124^I-labeled trastuzumab, have been screened to monitor HER2 levels in malignancies in preclinical studies (15–18). Although these probes showed good imaging properties, their slow tumor penetration and blood clearance results in imaging were performed several days after injection, which preclude clinical applications. Additionally, because of the long biological half-lives of ^89^Zr-trastuzumab and ^124^I-trastuzumab, the radiation-absorbed doses were calculated to be 0.5 mSv/MBq and 0.3011 ± 0.005 mSv/MBq, respectively, in patients, which were higher than those of ^18^F-FDG (0.019 mSv/MBq) (16, 17). As early as 2009, Orlova suggested that the use of the radionuclide-labeled affibody provides much better contrast in HER2 imaging than antibody due to the more rapid clearance from the blood and normal organs (19). The Z_HER2:342_ affibody is one of the most widely studied HER2 affibody molecules and is suitable for HER2 binding since it does not interfere with HER2-targeted therapy due to the use of different HER2 binding domains in the receptors (20). Several derivatives of Z_HER2:342_ have been developed and evaluated in preclinical and clinical Single photon emission CT (SPECT) and PET studies. ^18^F is preferred for clinical use due to its good imaging properties and commercial availability (21). [^18^F]AlF is rapidly expanding as an ^18^F labeling technique that allows convenient ^18^F labeling in less time and under milder conditions that can also be combined with a kit (22, 23).

Preclinical studies have shown that [^18^F]AlF-NOTA-MAL-M_ZHER2:342_ is a promising tracer for *in vivo* detecting HER2 status (24). Although 9.3% radiochemical yield is greatly improved on the previous ^18^F labeled Z_HER2:342_, it still has some limitations for clinical translation. It results in a high concentration of ^18^F to be used in radiosynthesis, which increases radiation exposure of technicians. So exploring improved derivatives of Z_HER2:342_ and effective labeling methods to increase the labeling yield deserves further study.

Thus, we hypothesized that the HER2 affibody molecules with the amino acid sequence apoptosis-enhancing nuclease (AEN)- at the N-terminus and NOTA-based chelators labeled with ^18^F using the new one-step labeling method, with preserved binding specificity to HER2, would yield a low abdominal background and would possess more favorable *in vivo* pharmacokinetic performance (25). The aim of the present study was to determine whether the [^18^F]AlF-NOTA-HER2 affibody is a promising imaging candidate for HER2 detection in GC.

## Materials and Methods

### Synthesis and Radiolabeling

All chemicals used in this study were purchased commercially. The HER2 affibody (AENKFNKEMRNAYWEIALLPNLNNQQKRAFIRSLYDDPSQSANLLA EAKKLNDAQ) was synthesized using manual solid-phase peptide synthesis. NOTA was coupled to the N-terminus of the HER2 affibody. The synthetic NOTA-HER2 affibody was obtained from China Peptides Company (China). Electrospray ionization-mass spectrometry (ESI-MS) was performed to analyze the identity of the final product. (HPLC) was used to analyze the purity of NOAT-HER2 affibody conjugate.

A QMA cartridge carrying ^18^F^−^ was pretreated with NaHCO_3_ (0.5 M, 10 ml). Then, ^18^F^−^ was eluted from the cartridge using 0.4 ml of saline. ^18^F^−^ in saline (0.1 ml, 111 MBq-962 MBq), potassium hydrogen phthalate (KHP) buffer (13 μl, 0.5 M), and AlCl_3_·6H_2_O (6 μl, 2 mM) in KHP buffer (0.05 M) were mixed and incubated at room temperature for 5 min. Then, 14 μl of HER2 affibody (20 nM) was added to the mixture and heated at 110°C for 15 min. After cooling to room temperature, the mixture was passed through a Sep-Pak C18-Light Cartridge that was activated with 10 ml of EtOH and H_2_O. The Sep-Pak C18-Light Cartridge was washed with 10 ml of H_2_O and extracted with 0.5 ml of 80% EtOH to obtain the final product.

### *In vitro* and *in vivo* Stability

The *in vitro* stability of the [^18^F]AlF-NOTA-HER2 affibody was determined in 5% human serum albumin (HSA) and physiological saline at 37°C. After 0, 1, 2, and 4 h, 20 μl of the mixture were analyzed using HPLC.

For the analysis of *in vivo* stability, 200 μl of [^18^F]AlF-NOTA-HER2 affibody (7.4 MBq) were intravenously injected into the caudal region of BALB/c nude mice. After 30 min, blood was collected and centrifuged, and the supernatant was analyzed using HPLC.

### Cell Culture

The NCI-N87 cell line derived from human gastric adenocarcinoma with high expression of HER2 was kindly provided by the Stem Cell Bank, Chinese Academy of Sciences. Adherent cells were cultured in RPMI 1640 Medium (Thermo Fisher Scientific, Boston, MA, USA) supplemented with 1% sodium pyruvate 100 mM solution and 10% fetal bovine serum (FBS) (BI, Kibbutz Beit-Haemek, Israel) at 37°C in an atmosphere containing 5% CO_2_. The GC cell line MKN74 (HER2-negative) was obtained from Otwo Biotech (Guangzhou, China) and cultured in RPMI-1640 medium supplemented with 10% FBS.

### Cellular Uptake, Retention Kinetics, and Blocking Studies

NCI-N87 and MKN74 cell lines were cultured in 24-well plates at a density of 1 × 10^5^ cells/well-overnight, and the fresh culture medium containing was replaced. Subsequently, the cells were incubated with the [^18^F]AlF-NOTA-HER2 affibody (111 KBq/well). After 5, 30, 60, and 120 min, the supernatants were collected, and the cells were washed twice using PBS. Radioactivity was measured using a γ-counter. The radioactive medium and PBS were defined as C_out_. Finally, the cells were harvested with trypsin and flushed again with PBS twice. Radioactivity counts of lysates and PBS were considered C_in_. The cellular uptake rate was calculated using the formula C_in_/(C_in_ + C_out_).

Cellular retention studies were performed in NCI-N87 and MKN74 cells. The cells were incubated with the [^18^F]AlF-NOTA-HER2 affibody (111 KBq/well) at 37°C for 1 h, and the culture medium was replaced by a fresh culture medium. After 5, 30, 60, and 120 min, the supernatants were collected, and the wells were washed with PBS. Counts containing radioactive supernatants and PBS were designated as C_out_. Then the cells were harvested with trypsin and flushed again with PBS twice. Radioactivity counts of lysis solution and PBS were defined as C_in_. The cellular retention ratio was calculated using the formula C_in_/(C_in_ + C_out_).

For the blocking assay, NCI-N87 cells were incubated with the [^18^F]AlF-NOTA-HER2 affibody in the presence of 6- and 35-fold excess unlabeled HER2 affibody for 60 min. The supernatant and precipitate were collected and analyzed as mentioned above.

### Saturation Binding Assay

The Kd value was studied by adding increasing concentrations (1.625–240 nM) of [^18^F]AlF-NOTA-HER2 to NCI-N87 cells. Excess unlabeled HER2 affibody (1,900 nM/well) was used as a blocking agent to determine the non-specific binding in another 48-well plate under the same treatment conditions. After removing the medium after 1 h of incubation, the cells were washed twice with PBS and detached with trypsin. The radioactivity of cells was measured and the Kd value was estimated using GraphPad Prism 5.

### Animal Model

All animal experiments were approved by the Principles of Ethical Committee of the Fourth Hospital Hebei Medical University (2018MEC123). Female BALB/c nude mice, 4 weeks old, were housed in ventilated filter-topped cages under specific pathogen-free (SPF) conditions with free access to a standard diet and water. Approximately 2 × 10^7^ gastric tumor cells (in 150 μl of Roswell Park Memorial Institute [RPMI] 1640 medium without FBS) were inoculated subcutaneously into the right forelimb to establish the tumor models.

### Biodistribution Studies

Female BALB/c mice-bearing NCI-N87 or MNK74 tumor xenografts were injected with the diluted [^18^F]AlF-NOTA-HER2 affibody (200 μl, 2.96 MBq) via the tail vein. The mice were sacrificed and dissected in groups (*n* = 3) at 30, 60, and 120 min postinjection (p.i.). Tumor, blood, and normal tissues were collected immediately after cleaning, weighed, and measured for radioactivity using a γ-counter after decay correction. The results are reported as the percentage of the injected dose per gram of tissue (%ID/g).

### Molecular Imaging Procedure

Mice-bearing NCI-N87 or MKN74 tumors (*n* = 6) were intravenously injected with the diluted [^18^F]AlF-NOTA-HER2 affibody (200 μl, 7.4 MBq). For blocking, 500 μg of cold NOTA-HER2 affibody were coinjected into each mouse bearing NCI-N87 tumors (*n* = 3). The images in mice-bearing NCI-N87 and MKN74 tumors were acquired at 30, 60, and 120 min p.i., and a blocking study was performed on NCI-N87 xenograft tumor-bearing mice. Imaging was performed using a digital Vereos PET/CT Scanner (PHILIPS, The Netherlands) acquired with 200 mm diameter Transaxial field of view (FOV) and ordered subsets expectation maximization (OSEM) + filtered back-projection (FBP) + time-of-flight (TOF) reconstruction algorithms. The images were digitally stored in a 1,024 × 1,024 matrix. The SUV_max_ values of the region of interest (ROI) over the tumor and muscle were collected.

At the same time, mice-bearing NCI-N87 tumors (*n* = 3) were intravenously injected with the diluted [^18^F]AlF-NOTA-HER2 affibody (200 μl, 7.4 MBq) and imaged by micro-PET/CT at 30, 60, and 120 min p.i. Another group of mice-bearing NCI-N87 tumors (*n* = 3) was intravenously injected with ^68^Ga-NOTA-HER2 affibody (200 μl, 7.4 MBq; *n* = 3) and imaged by micro-PET/CT at 30 and 60 min p.i.

### Statistical Analysis

All statistical analyses were completed using SPSS 22.0 software. Variable data are presented as the mean ± SD ($\bar{X}$ ± SD). The significance of differences in comparisons between two datasets was analyzed using Student's *t*-test. When three groups were compared, a one-way ANOVA was applied. A rank-sum test was also performed. A *p* < 0.05 was considered statistically significant.

## Results

### Synthetic Chemistry and Radiochemistry

**Figure 1:**
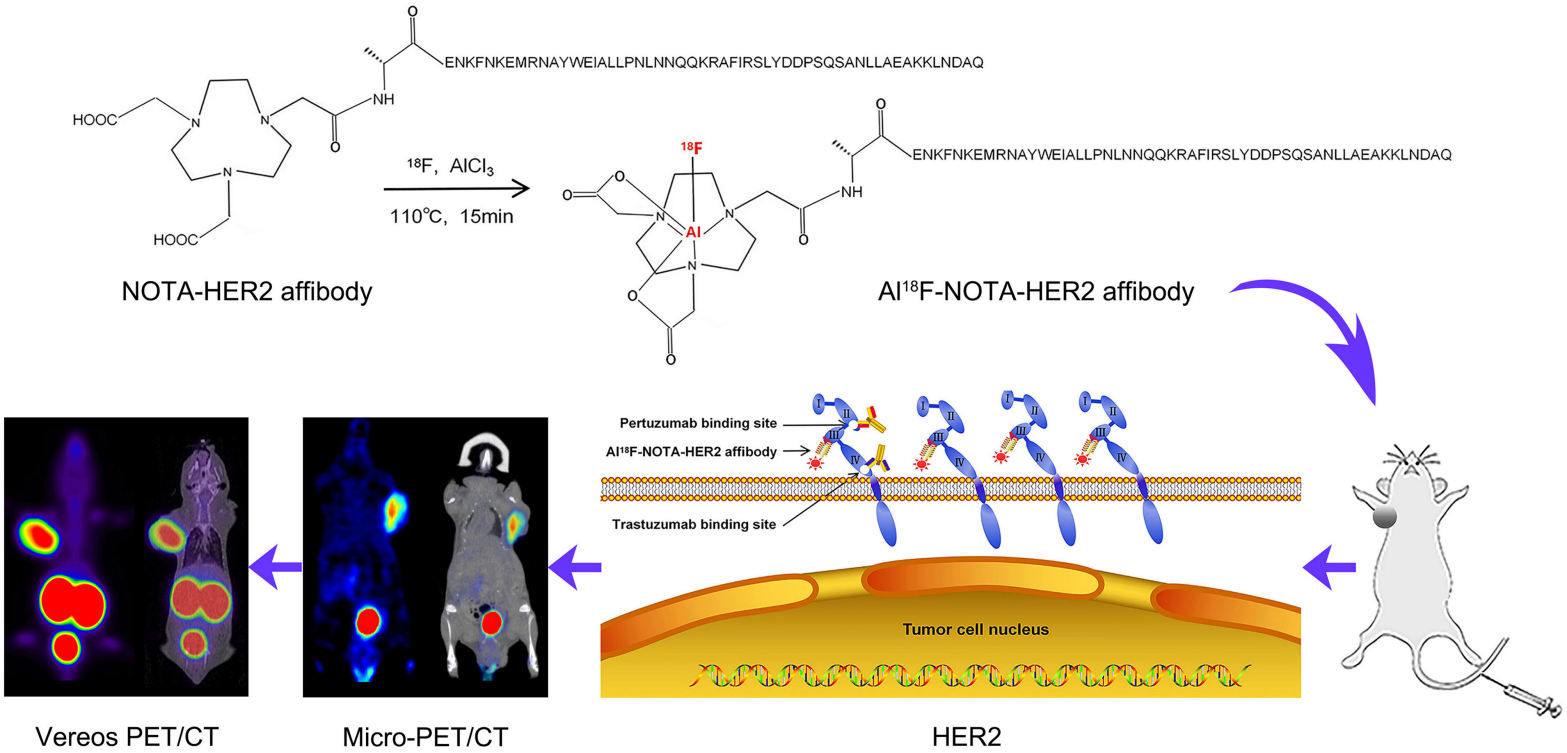
Drug synthesis and experimental route.

Alanine at the N-terminus of the HER2 affibody was subjected to a condensation reaction with -orcarboxylic acid (COOH) of NOTA [serving as a chelator for [^18^F]AlF labeling] (Figure 1). The purity of the obtained NOTA-modified affibody molecules was 97.80%. The MS spectrum indicated a molecular weight of 6680.51, which is consistent with its theoretical molecular weight.

**Figure 2:**
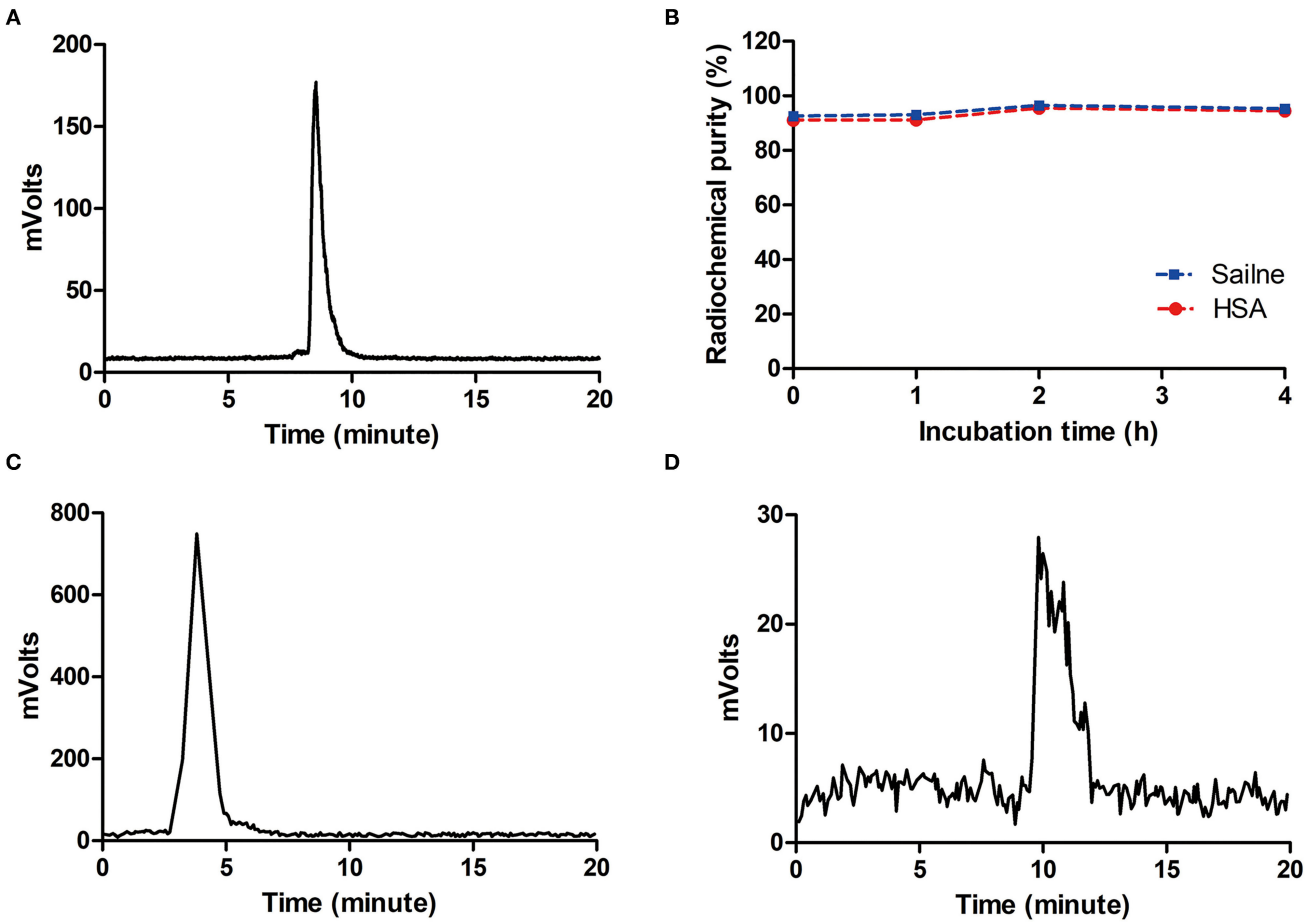
HPLC chromatograms of the [^18^F]AlF-NOTA-HER2 affibody **(A)** and ^18^F^−^ **(C)**. *In vitro* stability of the [^18^F]AlF-NOTA-HER2 affibody in saline and 5% HSA **(B)** and *in vivo* stability in blood for 30 min **(D)**. HPLC, high-pressure liquid chromatography; HAS, half-normal saline.

The [^18^F]AlF-NOTA-HER2 affibody molecular probe was manually prepared within 30 min. The maximum nondecayed corrected yield of the [^18^F]AlF-NOTA-HER2 affibody molecular probe was 32.69% with a radiochemical purity >98%, as analyzed by HPLC with a retention time of 8.5 min (Figure 2A).

### Stability

The radiochemical purity of the [^18^F]AlF-NOTA-HER2 affibody *in vitro* following an incubation with physiological saline or 5% half-normal saline (HAS; 37°C, 4 h) was maintained at >90% (Figure 2B), and a single peak was observed without free ^18^F^−^ (Figure 2C). The radiotracer was injected into mice, and a single peak was observed in blood at 30 min p.i. using HPLC, with a radiochemical purity >90% (Figure 2D). The results indicated that the [^18^F]AlF-NOTA-HER2 affibody was stable *in vitro* and *in vivo* over the period tested.

### Cellular Uptake, Retention, and Blocking Assay *in vitro*

**Figure 3:**
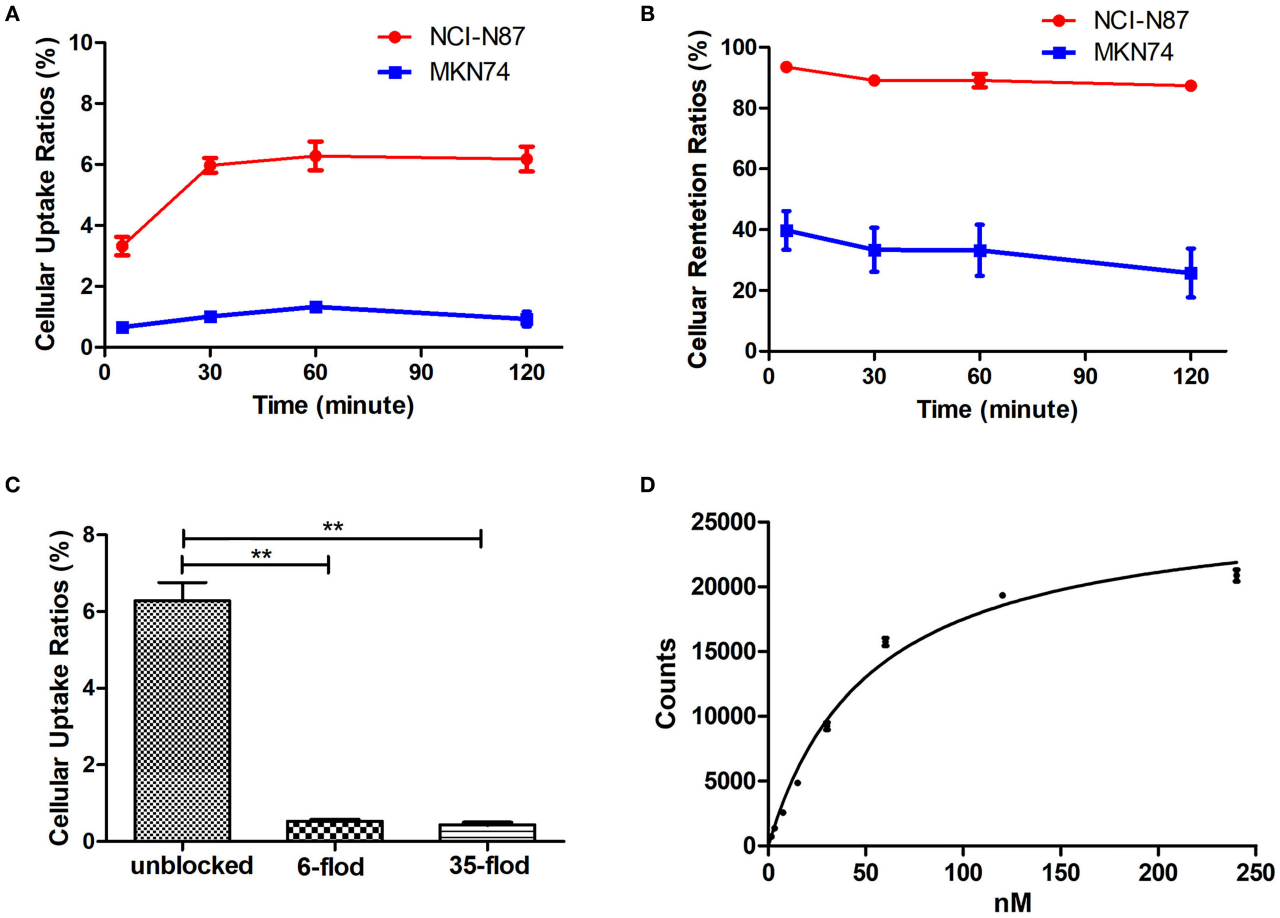
Cellular uptake **(A)**, and retention kinetics **(B)** of the [^18^F]AlF-NOTA-HER2 affibody in NCI-N87 and MKN74 cells. Blockade of the cellular uptake of the [^18^F]AlF-NOTA-HER2 affibody in NCI-N87 cells **(C)** (**p < 0.01). Binding saturation curve of the [^18^F]AlF-NOTA-HER2 affibody to HER2 in NCI-N87 cells **(D)**.

The [^18^F]AlF-NOTA-HER2 affibody showed higher cellular uptake and retention in NCI-N87 cells than in MKN74 cells at all time points tested (Figures 3A,B). Radioactivity accumulated rapidly in NCI-N87 cells, peaked at 60 min (6.28 ± 0.47%), and decreased slightly over time. Additionally, HER2-negative MKN74 cells did not show any significant uptake of the [^18^F]AlF-NOTA-HER2 affibody, suggesting the specific HER2-mediated uptake of the molecular probe in HER2+ cells.

The uptake of the [^18^F]AlF-NOTA-HER2 affibody in NCI-N87 cells was 6.28 ± 0.47% and was reduced to 0.53 ± 0.03% and 0.44 ± 0.05% by 6- and 35-fold excesses of cold HER2 affibody at 60 min, respectively (Figure 3C). The amount of [^18^F]AlF-NOTA-HER2 affibody taken up by NCI-N87 cells was decreased by more than 90% after an incubation with an excess of cold HER2 affibody, indicating that the uptake of the molecular probe in NCI-N87 cells was HER2 specific.

### Saturation Binding *in vitro*

The saturation binding curve of the Kd determination is shown in Figure 3D. The Kd value of the [^18^F]AlF-NOTA-HER2 affibody to HER2 in NCI-N87 cells was 52.25 ± 3.68 nM, indicating that the radiolabeled tracer binds to HER2 with high affinity and specificity.

### *Ex vivo* Distribution

**Figure 4:**
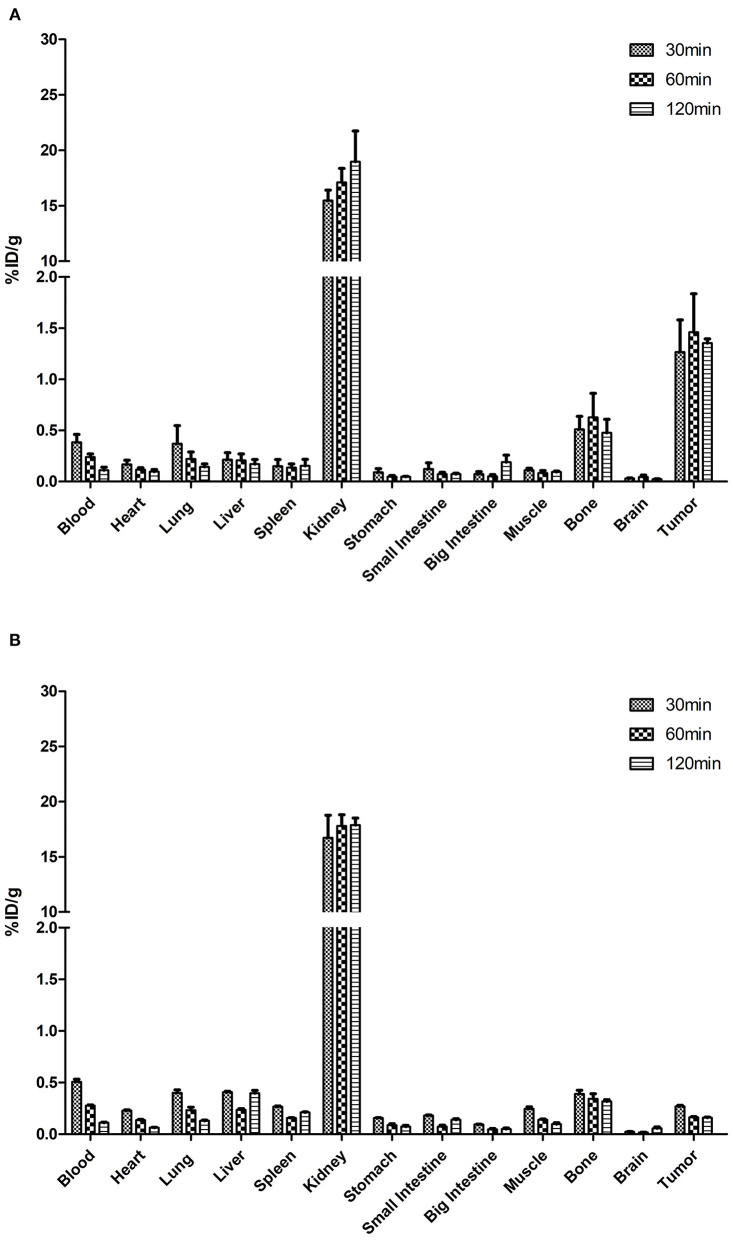
Biodistribution of the [^18^F]AlF-NOTA-HER2 affibody in mice-bearing NCI-N87 **(A)** and MKN74 **(B)** tumors.

The [^18^F]AlF-NOTA-HER2 affibody was efficiently accumulated in HER2-expressing tumors. HER2+ tumor uptake of the radiolabeled agent was 1.27 ± 0.54% ID/g, 1.46 ± 0.65% ID/g, and 1.35 ± 0.07% ID/g at 30, 60, and 120 min p.i., respectively (Table 1). The [^18^F]AlF-NOTA-HER2 affibody showed a significantly higher concentration in HER2+ tumors than in HER2– tumors at each time point (*p* = 0.003, 0.016, and 0.010, respectively; Figure 4), which was consistent with the tumor/muscle ratio (T/M) (*p* = 0.046, 0.046, and 0.002, respectively). The kidney showed the highest uptake of the radiotracer, suggesting that the urinary system is the main route of excretion.

**Table 1:** Biodistribution of the [^18^F]AlF-NOTA-HER2 affibody in mice-bearing NCI-N87 tumors.

**Organ (Mean ± SD, %ID/g)**	**30 min**	**60 min**	**120 min**
Blood	0.38 ± 0.14	0.24 ± 0.05	0.11 ± 0.05
Heart	0.17 ± 0.08	0.12 ± 0.03	0.10 ± 0.03
Lung	0.37 ± 0.31	0.22 ± 0.12	0.14 ± 0.05
Liver	0.21 ± 0.12	0.21 ± 0.11	0.17 ± 0.08
Spleen	0.15 ± 0.11	0.14 ± 0.06	0.15 ± 0.11
Kidney	15.48 ± 1.60	17.11 ± 2.17	18.97 ± 4.82
Stomach	0.09 ± 0.06	0.05 ± 0.02	0.05 ± 0.01
Small intestine	0.12 ± 0.10	0.07 ± 0.03	0.07 ± 0.02
Large intestine	0.07 ± 0.04	0.05 ± 0.03	0.19 ± 0.12
Muscle	0.11 ± 0.04	0.09 ± 0.04	0.09 ± 0.02
Bone	0.51 ± 0.22	0.63 ± 0.40	0.48 ± 0.23
Brain	0.03 ± 0.01	0.04 ± 0.03	0.03 ± 0.01
Tumor	1.27 ± 0.54	1.46 ± 0.65	1.35 ± 0.07

The [^18^F]AlF-NOTA-HER2 affibody concentration in the blood was decreased from 0.38 ± 0.14% ID/g at 30 min p.i. to 0.11 ± 0.05% ID/g at 120 min p.i. (Table 2). The radiolabeled affibody molecules showed rapid clearance from the blood and normal organs, causing a high tumor-to-background ratio.

**Table 2:** Biodistribution of the [^18^F]AlF-NOTA-HER2 affibody in mice-bearing MKN74 tumors.

**Organ (Mean ± SD, %ID/g)**	**30 min**	**60 min**	**120 min**
Blood	0.51 ± 0.04	0.28 ± 0.01	0.11 ± 0.01
Heart	0.23 ± 0.01	0.14 ± 0.01	0.07 ± 0.01
Lung	0.40 ± 0.05	0.24 ± 0.05	0.13 ± 0.01
Liver	0.41 ± 0.02	0.24 ± 0.02	0.40 ± 0.04
Spleen	0.27 ± 0.01	0.16 ± 0.01	0.21 ± 0.01
Kidney	16.72 ± 3.53	17.80 ± 1.75	17.88 ± 1.10
Stomach	0.15 ± 0.01	0.09 ± 0.03	0.07 ± 0.02
Small intestine	0.18 ± 0.01	0.07 ± 0.02	0.14 ± 0.02
Big intestine	0.09 ± 0.01	0.05 ± 0.02	0.05 ± 0.01
Muscle	0.25 ± 0.03	0.14 ± 0.01	0.10 ± 0.03
Bone	0.39 ± 0.06	0.34 ± 0.09	0.32 ± 0.04
Brain	0.03 ± 0.01	0.01 ± 0.01	0.06 ± 0.02
Tumor	0.27 ± 0.02	0.16 ± 0.01	0.16 ± 0.01

### PET Imaging

**Figure 5:**
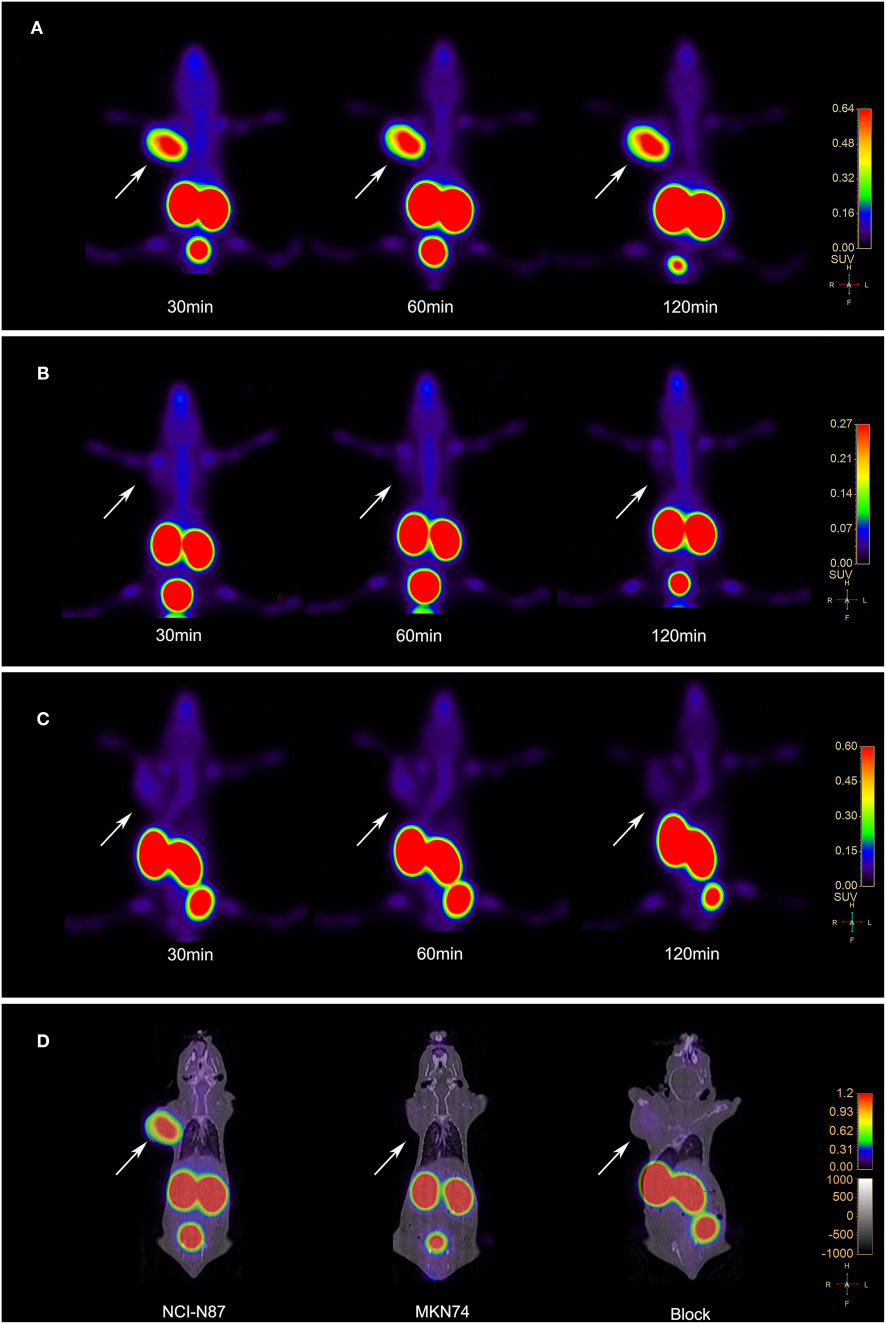
**(A)** Vereos PET images of the [^18^F]AlF-NOTA-HER2 affibody in tumor-bearing mice NCI-N87 xenografts. **(B)** Vereos PET images of the [^18^F]AlF-NOTA-HER2 affibody in tumor-bearing mice MKN74 xenografts. **(C)** Blocking study after a coinjection with excess cold NOTA-HER2 affibody in mice-bearing NCI-N87 tumors. **(D)** Fusion images of mouse model at 60 min post-injection by Vereos PET/CT. White arrows indicate xenografts.

**Figure 6:**
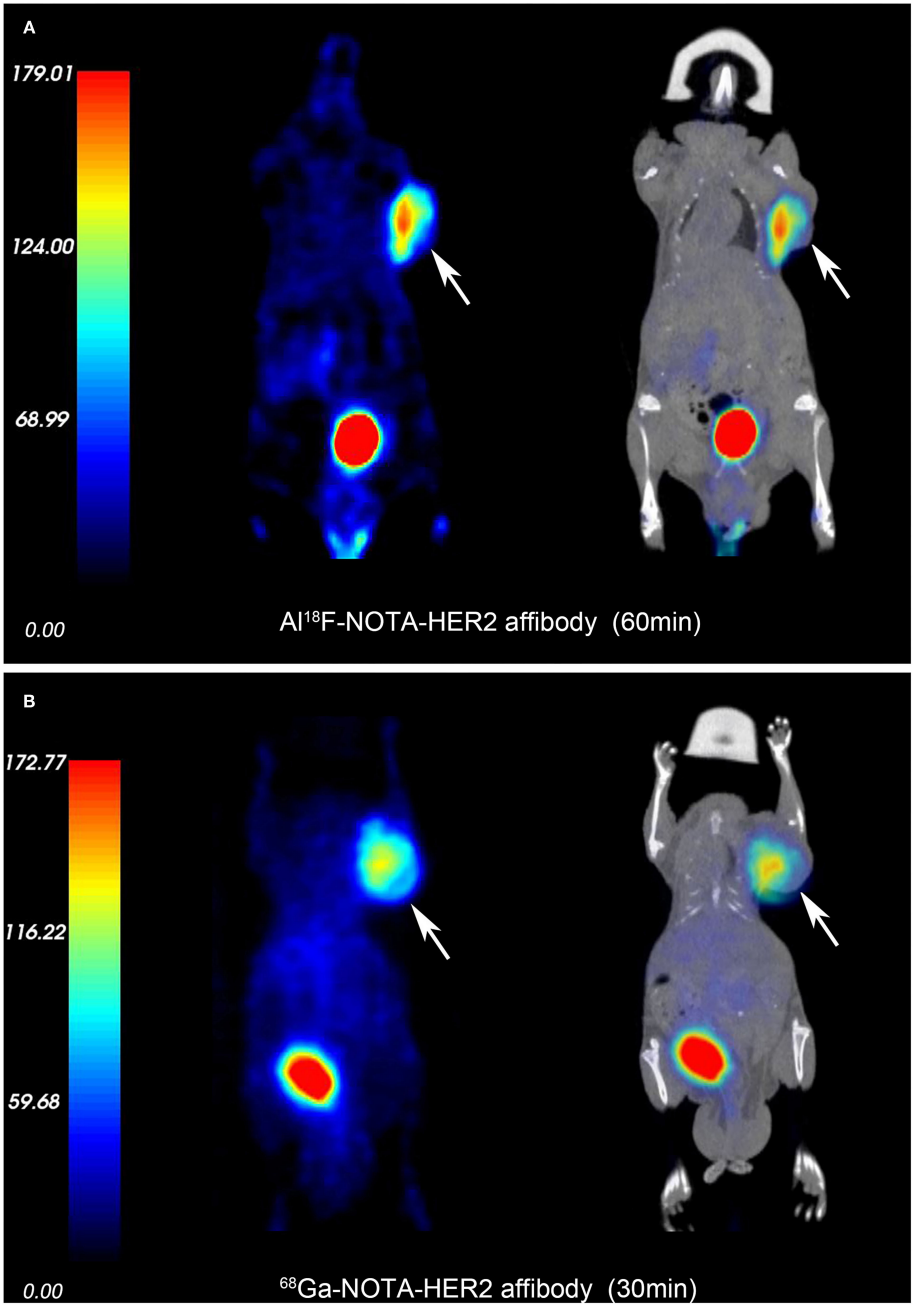
PET and fusion images of the [^18^F]AlF-NOTA-HER2 affibody **(A)** and ^68^Ga-NOTA-HER2 affibody **(B)** in mice-bearing NCI-N87 by micro-PET/CT. White arrows indicate xenografts.

3D-rendered digital Vereos PET/CT images of mice-bearing NCI-N87 and MKN74 tumors at 30, 60, and 120 min p.i. are displayed in Figure 5. As early as 30 min after injection, a considerably higher signal intensity was observed in HER2+ tumors and was consistently sustained at subsequent time points. HER2+ tumors, kidneys, and bladder were clearly visualized, while HER2– tumors were almost invisible, consistent with the biodistribution studies. The differences in T/M between NCI-N87 tumor-bearing mice and MKN74 tumor-bearing mice were statistically significant throughout the whole imaging process (*p* = 0.001, 0.004, and 0.004). The detection ability of Vereos PET/CT is comparable to that of micro-PET/CT in HER2+ tumors. In terms of image quality, the image resolution of [^18^F]AlF-NOTA-HER2 affibody is higher than that of ^68^Ga-NOTA-HER2 affibody (Figure 6).

The accumulation in NCI-N87 tumors was blocked by a coinjection of excess cold HER2 affibody at 30, 60, and 120 min after injection (Figure 5C). The quantitative analysis of T/M acquired from PET imaging data displayed a significant decrease in the blocked group compared to the non-blocked group (*p* = 0.003, 0.016, and 0.010).

## Discussion

The specific expression of HER2 in tumor biopsy samples is crucial in tumor diagnosis and the selection of the appropriate therapy. This approach may not be reliable for gastric tumors, which display high HER2 heterogeneity. Molecular imaging has great potential to characterize important specific targets in malignancies, such as HER2. Moreover, ^68^Ga-labeled affibody probes have been reported to be successful in many preclinical investigations and a first-in-human study in HER2+ tumor imaging (26, 27). However, applications of the ^68^Ga labeling method are limited due to the relatively short half-life (67.7 min) of ^68^Ga compared with ^18^F.^18^F-labeled affibody molecules that meet the requirements for clinical use with a single good manufacturing practice production and a longer time window for image acquisition. The positron energy of ^18^F is lower than ^68^Ga (E_mean_ = 0.25 MeV vs. 0.83 MeV), resulting in a shorter positron range than ^68^Ga (0.6 vs. 3.5 mm), which leads to a higher spatial resolution in PET/CT imaging (28). The high image resolution of ^18^F and easy formation of [^18^F]AlF chelates make [^18^F]AlF molecular probes an engaging approach (29–32). Inspired by the emergence of [^18^F]AlF labeling strategy and the fact that [^18^F]AlF-NOTA-Octreotide is developed as a clinical substitute for ^68^Ga labeled somatostatin analog, we constructed a promising HER2-targeting tracer, the [^18^F]AlF-NOTA-HER2 affibody, and evaluated the biodistribution characteristics and preliminary HER2-targeted imaging efficacy in animal models. Meanwhile, we compared the image quality between [^18^F]AlF-NOTA-HER2 affibody and ^68^Ga-NOTA-HER2 affibody.

The preparation of [^18^F]AlF-NOTA-Z_HER2:342_ and PET imaging study have already been reported previously (24). However, compared with previous studies, the labeling rate of [^18^F]AlF-NOTA-HER2 affibody in our study has been greatly improved. Using KHP as the reaction buffer, which was different from sodium acetate buffer, the volume of the reaction system was reduced, and the labeling rate was increased to a certain extent compared to sodium acetate buffer in the previous study (23, 29, 30), which allows a lower concentration of ^18^F to be used in radiosynthesis. Moreover, KHP with non-volatile characteristics is suitable for the preparation and automatic production of kits, which lays the foundation for clinical translation in the future.

The [^18^F]AlF-NOTA-HER2 affibody with a Kd value of 52.25 ± 3.68 nM showed good uptake in the HER2+ tumor cell, and the *in vitro* uptake was blocked by excess cold HER2 affibody, indicating that the probe had high target-binding affinity and specificity in HER2+ cells. The high uptake of [^18^F]AlF-NOTA-HER2 affibody by HER2+ cells led to an obvious increase in the brightness of HER2+ xenografts on PET images. NCI-N87 xenografts were clearly distinguished from the surrounding normal organs at 30–120 min p.i. Quantification of PET imaging showed that high T/M ratios (12.19 ± 4.71) persisted at 120 min after injection, consistent with distribution study. Meanwhile, the coinjection of excess HER2 affibody noticeably decreased NCI-N87 tumor accumulation. These results confirmed the excellent HER2 targeting specificity *in vivo*. Moreover, the suitable half-life of ^18^F allows a longer time window for [^18^F]AlF-NOTA-HER2 affibody imaging acquisition, which is more practical in clinical situations. In order to clarify the advantage to use [^18^F]AlF, we contrasted PET images between [^18^F]AlF-NOTA-HER2 affibody and ^68^Ga-NOTA-HER2 affibody. The results demonstrated that [^18^F]AlF-NOTA-HER2 affibody PET can provide higher image resolution than that of ^68^Ga-NOTA-HER2 affibody. Digital Vereos PET/CT can provide high-quality images and the tumor detection ability of it is comparable to that of micro-PET/CT, which will widen the road for the development of preclinical experimental in some institutions without micro-PET/CT.

The biodistribution results were consistent with the quantitative analysis of PET data, and the [^18^F]AlF-NOTA-HER2 affibody was rapidly localized in NCI-N87 xenografts and was quickly cleared from the blood. As an improved structure of Z_HER2:342_, the HER2 affibody used in this study contains the amino acid sequence AEN- at the N-terminus, which might be associated with low hepatic uptake. The probe was removed from blood circulation predominantly by urinary excretion resulting in optimized contrast and improved imaging sensitivity and accuracy.

No free ^18^F^−^ or other dissociated products were detected in the stability study, indicating that the [^18^F]AlF-NOTA-HER2 affibody was highly stable *in vitro* and *in vivo*. Although the biodistribution results showed that the radioactive accumulation was slightly higher in bone than in most non-target organs, the low radioactive uptake of bone observed in PET images did not affect tumor detection. PET imaging revealed high HER2+ tumor-to-bone contrast, indicating that [^18^F]AlF was stably complexed to the HER2 affibody by NOTA. Nonetheless, it should be considered that bone accumulation of the free ^18^F^−^ might hamper visualization of small lesions or lesions with low HER2 expression in the clinical setting. Whether minor interference originating from ^18^F^−^ accumulation in the bone affects the detection of bone metastases remains to be verified in the next clinical trials.

The combination therapy of chemotherapy and trastuzumab was approved as the first-line treatment for HER2-positive AGC by the FDA in 2010 (16). Many studies have shown that radiolabeled trastuzumab can efficiently detect HER2. The trastuzumab-based probe has some disadvantages in monitoring changes in HER2 expression during trastuzumab treatment because the therapeutic antibody might compete with the probe for the same epitope on HER2 (27, 33). So we deduced that ongoing HER2-targeted therapy did not interfere with [^18^F]AlF-NOTA-HER2 affibody imaging, because the affibody and trastuzumab bind to different HER2 domains. Whether the [^18^F]AlF-NOTA-HER2 affibody can be used to monitor the initial response to treatment with trastuzumab and to re-evaluate the systemic HER2 status during trastuzumab treatment need further research.

This research also has some limitations. The radiation-absorbed doses of the kidney are high, renal protection could be performed by the use of positively charged amino acids, albumin fragments, or gelofusine. Moreover, the labeling rate needs to be further improved to minimize radiation exposure for technicians by subtly adjusting the ratio of HER2 affibody to AlCl_3_·6H_2_O solution or the pH value of the reaction system.

## Conclusions

The [^18^F]AlF-NOTA-HER2 affibody can be produced in high yields and with high radiochemical purity in a one-step procedure within 30 min. This new radiolabeled tracer can visualize HER2+ tumors with high specific tumor uptake and rapid blood clearance. [^18^F]AlF-NOTA-HER2 affibody may serve as a novel PET molecular probe with tremendous clinical potential for the non-invasive, real-time, and whole-body detection of the HER2 status in GC with good image contrast and resolution. This method could provide an *in vivo* understanding of GC biology that will ultimately guide the accurate diagnosis and treatment of GC. It is worth the clinical transformation.

## Data Availability Statement

The raw data supporting the conclusions of this article will be made available by the authors, without undue reservation.

## Ethics Statement

The animal study was reviewed and approved by the Principles of Ethical Committee of the Fourth Hospital Hebei Medical University (2018MEC123).

## Author Contributions

JH, YC, YZ, XZ, JZ, JW, and ZZ contributed to the study conception and design. Material preparation, data collection, and analysis were performed by JH and YC. The first draft of the manuscript was written by JH. The manuscript was reviewed by XZ. All authors commented on previous versions of the manuscript, contributed to the article, and approved the submitted version.

## Funding

This work was financially supported by grants from the National Natural Science Foundation of China (NSFC) project (Grant No. 82071959) and Hebei Provincial Natural Science Foundation, Jing-Jin-Ji special projects for basic research cooperation (Grant No. H2018206600).

## Conflict of Interest

The authors declare that the research was conducted in the absence of any commercial or financial relationships that could be construed as a potential conflict of interest.

## Publisher's Note

All claims expressed in this article are solely those of the authors and do not necessarily represent those of their affiliated organizations, or those of the publisher, the editors and the reviewers. Any product that may be evaluated in this article, or claim that may be made by its manufacturer, is not guaranteed or endorsed by the publisher.
